# Understanding progression of strictures in ileal Crohn's disease—The importance of setting methodological standards

**DOI:** 10.1002/ueg2.12327

**Published:** 2022-10-17

**Authors:** Helena Tavares de Sousa, Fátima Carneiro

**Affiliations:** ^1^ Gastroenterology Department Algarve University Hospital Center Portimão Portugal; ^2^ ABC—Algarve Biomedical Center University of Algarve Faro Portugal; ^3^ Department of Pathology São João University Hospital Center and Faculty of Medicine University of Porto Porto Portugal; ^4^ Institute of Molecular Pathology and Immunology of the University of Porto (Ipatimup) Porto Portugal; ^5^ Institute of Investigation and Innovation in Health (i3S) University of Porto Porto Portugal

Crohn's disease (CD) is a chronic inflammatory bowel disease most frequently involving the terminal ileum and right colon. Given its transmural nature, CD can lead to progressive bowel damage and complications (e.g., strictures, fistulas and abscesses).^1^ Over 10% of patients present strictures at diagnosis,^2^ with 15% to over 20% of the remainders developing them through the next 10 and 20 years, respectively.^3^ Importantly, strictures coexist in over 85% of penetrating CD.^4^ Population‐based studies showed a 10‐year cumulative risk of surgery (due to stricturing and/or penetrating complications) between 40% and 71%.^5^ After intestinal resection, anastomotic recurrence is the rule, leading to re‐stricturing and need of intervention, either through endoscopic balloon dilation (EBD) or surgery.^6^ Despite the high rates of stricturing disease, a gap remains in the understanding of the risk factors and progression rate for stricture development, which are crucial for patient risk stratification and selection of those benefiting the most from intervention. Noteworthy, prior studies on stricturing CD presented significant methodological caveats: (1) lack of a standard definition of stricture, (2) mixed populations (stricturing disease with and without associated fistulae, ileal and colonic strictures, anastomotic and primary strictures), (3) variable, non‐validated outcomes (subjective obstructive symptoms, different success criteria for therapeutic interventions), and (4) different imaging modalities and protocols, overall contributing for heterogeneous results.

The study by El Ouali et al.^7^ aimed to address many of these gaps in research. The authors included only patients with a strictly‐defined “non‐penetrating terminal ileum (TI) stricturing CD”—as per the CrOhN's disease anti‐fibrotic STRICTure therapies [CONSTRICT] group criteria,^8^ treated according to current standard of care. Additionally, they implemented centrally‐read (blinded to outcome measures) and state‐of‐art protocolised‐MRE, and rated obstructive symptoms through an own‐devised 7‐point index. The study included a derivation‐cohort from which a predictor model was built and a subsequent validation‐cohort that validated the model and its predictors. With this stringent methodology, El Ouali et al.^7^ could establish the rate of progression to, the risk factors for, and the need of intervention (EBD or surgery) in “pure” TI stricturing CD. Intervention rates at 12, 24 and 48 months were 26%, 35% and 45%, respectively. Importantly, shorter duration (HR 0.97 [0.95–0.995], *p* = 0.016) and increased length (HR 1.04 [1.01–1.07], *p* = 0.007) of the stricture, together with higher obstructive index (HR 1.44 [1.13–1.85], *p* = 0.004) were validated as predictors of subsequent intervention. On univariate analyses, an anastomotic stricture associated with EBD (HR 8.10 [1.02–64.16]; *p* = 0.047) while decision for surgery associated strongly with restricted diffusion on MRE (HR 10.62 [1.24–91.13]; *p* = 0.03), followed by past smoking (HR 3.75 [1.31–10.77]; *p* = 0.01), nausea/vomiting (HR 2.62 [1.03–6.67]; *p* = 0.04), obstructive index (HR 1.41 [1.07–1.87]; *p* = 0.02) and stricture length (HR 1.04 [1.01–1.07]; *p* = 0.003).

This work raises important issues to clinical practice. First, almost half of the patients required intervention (surgery or EBD) at 48 months of follow up. This might seem to challenge data from previous studies where anti‐TNFα treatment prevented surgery in over half of patients with symptomatic stricturing disease at 40–48 months^9^, ^10^ Yet, only 26%^10^ or 29%^9^ of these patients maintained successful response to anti‐TNFα (with no add‐on medical or endoscopic therapy) at 40 and 48 months, respectively, even if only early disease (median disease duration 2.9 years [0.6–8.6]), biologic‐naïve, non‐operated patients were included.^10^ These data, together with the fact that in this cohort, biologic use did not impact the need for intervention, reinforce the notion that these agents do not alter the natural history of the disease since fibrosis and inflammation always coexist^11^ and the first cannot be reversed with current biologic therapy.^4^ Second, although 40% of the included patients were asymptomatic at baseline, almost half of them developed obstructive symptoms after 48 months. Clinicians must be aware of the disconnect between symptoms and progression of disease and recognise the impact of the herein identified predictors of intervention. Third, despite some limitations (single‐center, observational, retrospective study, including a limited (*n* = 86) number of patients), this work has the potential to have an impact both in clinical practice and in clinical trials. Remarkably, the article provides an accessible online risk calculator for predicting intervention. Future studies should reproduce these findings prospectively and in non‐quaternary centers, providing a real‐life model validation.

Finally, El Ouali et al.’s study^7^ confirms the importance of setting standards for defining study populations and imaging methodology when addressing strictures or their development in CD. This article can be integrated in the enormous effort that has been put by the Stenosis Therapy and Anti‐fibrosis Research Consortium [STAR] for several years, in developing endpoints^8^ and standardized methodology for clinical,^12^ radiologic^13^ and histopathologic scoring systems,^14^ in order to build up the much needed clinical trials with antifibrotic agents.^15^

## CONFLICT OF INTEREST

The author declares that there is no conflict of interest that could be perceived as prejudicing the impartiality of the research reported.

## Data Availability

The data mentioned in this text can be found in the corresponding reference.
